# Functional Understanding of Quantum Technologies is Essential to the Ethical Debate About Their Impact

**DOI:** 10.1007/s11569-026-00501-7

**Published:** 2026-03-06

**Authors:** Eline de Jong

**Affiliations:** Institute for Logic, Language and Computation, Institute of Physics, Qusoft Research Center for Quantum Software, University of Amsterdam, Science Park 107, Amsterdam, 1098 XG The Netherlands

**Keywords:** Technological understanding, Functional understanding, Quantum technologies, Ethics, Societal impact, Capacity building

## Abstract

As the innovative potential of quantum technologies comes into focus, so too does the urgent need to address their ethical implications. While many voices highlight the importance of ethical engagement, less attention has been paid to the conditions that make such engagement possible. In this article, I argue that one key condition is *technological understanding*: the cognitive skill to recognise how a technology can be used to realise an aim. More specifically, I claim that meaningful ethical discussion depends, first and foremost, on understanding a technology’s functional capabilities: what it can do and what it can be used for. Presenting such ‘functional understanding’ as an epistemic requirement helps guide efforts to improve understanding of quantum technologies in support of ethical engagement. Such efforts often focus on explaining, in broad strokes, the underlying *physics* and technical details. While technical insights may indeed support ethical analysis, they are neither sufficient nor always necessary for grasping the broader societal and ethical implications of a technology. What is indispensable for such discussions is an understanding of its (potential) *functions.* To foster ethical engagement with quantum technologies, I therefore advocate a functions-oriented approach to promoting understanding of these technologies. At first glance, presenting technological understanding as an epistemic requirement for meaningful ethical engagement may seem to raise the bar for participation. However, by decoupling functional understanding from technical expertise, this condition becomes attainable for a broader group, contributing not only to a well-informed but also to a more inclusive ethical debate.

## Introduction

Quantum technologies represent a new frontier in technological advancement, promising to offer capabilities that surpass many classical physics-based technologies. This potential, often termed “the quantum advantage”, is associated with a host of opportunities and risks. Consequently, there have been numerous calls to scrutinise the ethical and broader societal aspects of these technologies while they can still be effectively shaped [1–7].

Although the calls for ethical engagement with quantum technologies have been loud and numerous, less attention has been given to the conditions that enable such engagement. Selin et al. [8] argue that effective (public) engagement depends on a range of *capacities*—specifically, the knowledge and skills required for meaningful participation in discussions of science and technology. I argue that one key capacity is ‘understanding’. Building on the work of De Jong and De Haro [9, 10], I propose technological understanding—i.e. recognising how a technology can be used to achieve a practical aim—as a foundational competence necessary for engaging in public discussions about technology. More specifically, I contend that technological understanding is a prerequisite for meaningful ethical debate about the impact of any technology.

However, given quantum’s reputation as being “too complex to understand”, this may raise concerns about the possibilities for and accessibility of meaningful ethical discussion. In this article, I address these concerns by mobilising a contextual account of understanding, which distinguishes different types of understanding, each relevant to a specific context. To foster ethical discussion about quantum technologies, I advocate for promoting a specific kind of technological understanding—one focused on the capabilities of quantum technologies and their potential practical deployment, rather than their underlying physics.

First, I explain why an understanding of the technology at hand is crucial to scrutinising its ethical aspects. Second, I draw on De Jong and De Haro’s account of technological understanding to clarify what such understanding amounts to, and discuss the three types of understanding that they distinguish. Third, I argue that it is, first and foremost, an understanding of quantum technologies’ functional capabilities—what they can do and be used for—that is essential for a meaningful ethical discussion. In doing so, I demonstrate the practical usefulness of De Jong and De Haro’s account and show why distinguishing between different types of technological understanding matters.

By presenting technological understanding as a precondition for ethical discussion, this article raises an epistemic threshold for ethical discussion of emerging technologies like quantum, emphasising the need to understand what a technology can do. But, by stressing the importance of understanding its functions rather than its underlying physics, it also makes this condition attainable for a wider audience, fostering an inclusive ethical debate.

## Understanding as a Critical Enabler of Ethical Enquiry

Imagine a blue device, small enough to hold in your hand, with two buttons labelled “read” and “write”. Would you feel sufficiently informed to discuss the ethical implications of this reader-writer? If I told you it was a “duplicator”, you would still lack essential information—what exactly does it duplicate? Now, suppose I reveal that this device is designed to read electronic data from a car key and write it into a new key. These details are crucial to engage in a well-informed or simply meaningful discussion about ethical concerns the duplicator brings about.

A certain degree of knowledge about a technology seems indispensable for critical thinking about its ethical aspects and broader societal impact, without the need to be a technical expert. Yet, knowledge alone is not enough; it must be combined with the ability to analyse and interpret its implications. This aligns with Selin et al. [8] who argue that effective public engagement with science and technology relies on (building) a range of “capacities*”*. These capacities build on both knowledge and analytical skills. Drawing on these considerations, I propose that understanding—as the ability to *use knowledge*—is a key enabler of meaningful ethical engagement.

In the philosophical literature, understanding has typically been conceived as *the ability to do something with knowledge* [11–15]. Some have further specified it as the ability to make counterfactual inferences: to reason about “what-would-happen-if” scenarios (e.g., [10, 11, 16]). When mapping ethical issues regarding the potential impact of some (future) technology, such inferential abilities play a crucial role. In other words, ethical engagement can be considered a cognitive task, requiring the ability to use knowledge about the technology to construct “what-if" scenarios (“what if the technology materialises in a specific way?”) and make “if-then” inferences (“if the technology has these capabilities, then those applications are possible”).

The idea of understanding as an enabling skill for ethical discussion resonates with recent calls to build understanding of quantum technologies ([1]: p.292, [3]: p.xix, [6, 17, 18]: p.40, [19]: p.3, [20, 21]). Vermaas [21], for example, argues that understanding quantum technologies to “a reasonable degree” is a prerequisite to a societal debate about them. Yet such calls often leave unspecified what this discussion-enabling understanding actually involves and what kind of capacity we need to build. What type or level of understanding of quantum technologies is necessary to consider their ethical aspects?

## Three Types of Technological Understanding

To determine which type of understanding of quantum technologies is relevant for ethical discussions, it is helpful to first clarify what it means to understand a technology at all. De Jong and De Haro [9, 10] address this in their account of “technological understanding”. They define it as a cognitive skill that consists *in the ability to (recognise how to)*^1^ *use a technology to realise a particular aim*. Applied to, for example, quantum computing, technological understanding thus means: being able to use a quantum computer to achieve a practical goal.

This ability implies that the technology must be intelligible to the user. A technology that is entirely opaque to its user cannot be meaningfully used to achieve an aim. To use a technology successfully, one must be able to reason prospectively about the consequences of operating it—about what will likely happen if it is used in a certain way. This involves grasping the technology’s properties and qualities, which in turn can be understood at different levels: technical, practical, or more conceptual.

Technological understanding thus requires some insight into how the technology works and what it can do. This is not an all-or-nothing matter, but a cognitive skill that comes in degrees. The required kind and depth of insight depend on the context in which an agent engages with the technology and on the goal being pursued. In this sense, what counts as (sufficient) technological understanding is context-dependent.

De Jong and De Haro distinguish three main contexts that involve technological understanding: the context of design, operation and innovation [9, 10]. In each of these contexts—which may overlap in practice—technological understanding is specified in a different way. This yields three contextual *types* of understanding, each foregrounding different aspects of the technology and involving different levels of intelligibility and reasoning. I briefly discuss each of these contexts and their associated types of technological understanding.

In a design context, where the goal is to outline and build a technology, technological understanding involves reasoning about the artefact at the level of its inner workings. To design any technology—from a microwave oven to a quantum network—designers need an understanding of an artefact’s principles and components in order to design a functioning system. In the case of a quantum computer, this ‘technical’ understanding consists in recognising how quantum phenomena such as superposition and entanglement can be controlled and manipulated in order to perform computational tasks.

Such technical understanding is not necessarily required in the operation context, where the focus is on practical use. Here, technological understanding requires recognising the direct consequences of interacting with the technology. A driver, for example, must understand how pressing the pedals or using the gear stick affects the car’s movement, without needing to comprehend the engine’s mechanics. Similarly, such ‘operational’ understanding of a quantum computer involves knowing how to practically operate it and successfully perform tasks with it, rather than understanding its internal physical and technical mechanisms.

The innovation context has yet another focus. Here, the central aim is to invent a new relationship between a technology and a practical aim. While design involves “thinking up” the artefact itself, innovation, as defined by De Jong and De Haro, entails thinking up its instrumentalisation: determining what a technology can be used for*.* This includes both repurposing existing technologies—for example, leveraging the capabilities of graphic processing units (GPUs) to train artificial neural networks—and identifying new problems or needs that could be addressed by a yet-to-be-designed technology.

Innovation-type understanding is typically involved in devising a technology’s applications: if researchers and engineers develop a large-scale quantum computer, what practical purposes could it serve? Answering that question requires recognising a technology’s *functional capabilities*. That is, understanding the principles that determine what kinds of tasks a technology enables, and where its limits lie. Importantly, this is not the same as superficial technical knowledge; it requires substantive insight into the aims for which the technology may be deployed.

For example, recognising the functional capabilities of a large language model (LLM) involves understanding that such models recognise patterns in data and generate text by probabilistically predicting plausible next words. This makes LLMs suitable for some tasks, but unreliable for others.

‘Functional’ understanding focuses on what the technology *can be expected to do*, rather than how its internal architecture is structured or how exactly it is operated, and thereby enables assessment of its fitness for purpose. In the case of a quantum computer it consists in recognising what it may plausibly be used for (e.g. breaking current encryption methods or speeding up aspects of drug design), as well as what it is not expected to do (e.g. provide an all-purpose computational speed-up over classical computers or automatically “supercharge” artificial intelligence).

I will argue that it is this functional understanding of (quantum) technology that is required for a meaningful ethical discussion about its implications.

**Fig. 1:**
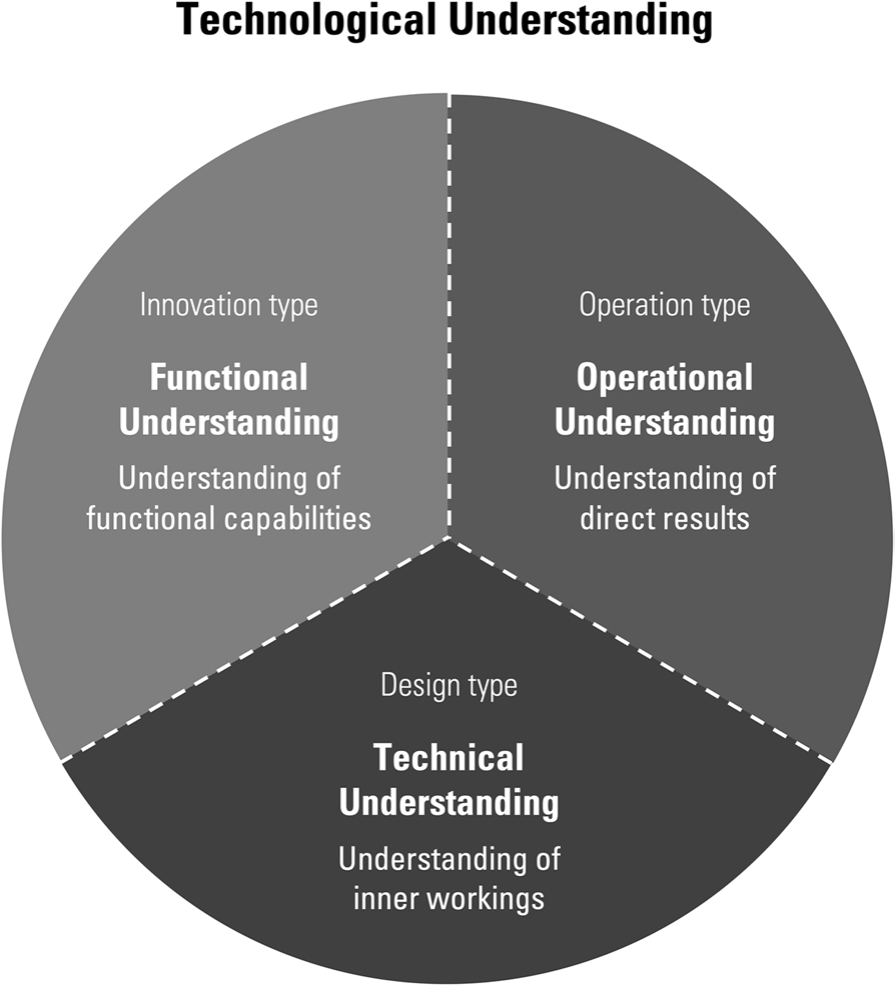
Three Types of Technological Understanding, each Associated with a Specific Context and Level of Understanding

Figure 1 summarises the three types of technological understanding.

As emphasised earlier, the three types of technological understanding are not mutually exclusive: a user may invent a new application, designers may be skilled users, and innovators may participate in the design process. Distinguishing between them highlights the different ways technological understanding can take shape, foregrounding certain aspects while backgrounding others. In the next section, I draw the practical implications of this contextual account by linking ethical engagement to one specific type of understanding.

## Understanding Functions rather than Physics

Thus far, I have argued that understanding is a key-enabling capacity for ethical engagement (Section “Understanding as a Critical Enabler of Ethical Enquiry”) and proposed technological understanding as the relevant kind (Section “Three Types of Technological Understanding”). Building on this, I will now argue that ethical discussions about quantum technologies—particularly those focused on assessing impact—require, first and foremost, the *functional* type of technological understanding: understanding what they can do and what they can be used for.

Recall the duplicator—in order to identify the ethical concerns it raises, it is crucial to understand what it actually *does*. Similarly, in ethical discussions about a quantum computer, what matters is understanding its capabilities and the ways it might be used. Assessing the potential impact of quantum technologies thus requires envisioning their possible applications. This ability aligns primarily with functional understanding, which entails grasping a technology’s capabilities and the range of uses they enable. In short, ethical engagement with quantum technologies requires an understanding of their potential *functions*.

In practice, however, efforts aimed at fostering public understanding of quantum technologies often tend to focus on promoting a general understanding of its underlying *physics.* An empirical study by Van de Merbel et al. [22] found that 81% of Dutch news articles about quantum technologies explain the physics behind quantum technologies. In the same study, the ability to recognise quantum phenomena is taken as an indicator of knowledge about quantum science and technology [22]. Another study found that, in 70% of the TEDx talks given by experts about quantum science and technology, one or more quantum phenomena are explained [23]. Introducing quantum technologies to a broader audience thus often involves an introduction to quantum mechanics.

This also aligns with my experience over recent years, where introductions to quantum technologies in public-facing and interdisciplinary contexts often focus on explaining the underlying physics. Such introductions are typically not highly technical or mathematically detailed, but instead offer schematic explanations of core quantum phenomena (such as superposition or entanglement) as a way of making quantum technologies intelligible to non-specialist audiences. One plausible explanation for this is the early stage of the field: with its capabilities still largely conceptual and many applications still uncertain, the underlying physics often serves as the primary point of introduction.

This trend is also visible in academic and policy discussions: in this emerging field, many publications on the ethical and societal aspects of quantum technologies include sections on quantum phenomena such as superposition and entanglement [3, 5, 19, 21, 24–26]. These patterns reveal a ‘physics-first approach’ to fostering understanding of quantum technologies in which scientific and technical explanations are treated as the primary route to engage people beyond physics communities. This tendency is illustrated by Vermaas’ specific call to philosophers of physics “for a renewed effort to make quantum theory understandable” in order to equip a broader group of stakeholders to engage in the societal debate on quantum technologies, suggesting that their expertise uniquely positions them to improve understanding among a wider audience ([21]: p.242).

While there is variation in the degree of technical detail, a physics-first approach consistently foregrounds technical explanations to promote understanding of quantum technologies. The issue is less with the ‘physics’ and more with the ‘first’. Basic explanations of quantum mechanics can spark public interest [17, 27] and provide a useful backdrop for those who are curious. However, they generally do not make quantum technologies epistemically accessible in a way that enables meaningful ethical discussion. After all, most people do not fully understand how combustion engines, transistors, or GPUs work, yet they can still engage in ethical debates about cars, smartphones, and AI. Likewise, explanations of quantum phenomena, quantum circuits, quantum gates, and quantum bits (‘qubits’) are of limited help when exploring the potential impact of quantum computing: at best, they offer a vague sense of what makes quantum computing different, but they rarely equip people to understand how those differences translate into societal or ethical implications. To explore such implications, it is crucial to develop a sense of the kinds of tasks a quantum computer promises to perform. While these capabilities are ultimately rooted in technical details, it is possible, to a large extent, to develop functional understanding of them without first acquiring technical understanding.

Mentioning ‘superposition’ in discussions of a quantum computer is, of course, not a crime; it would be odd to omit it entirely. However, focusing too heavily on technical explanations risks misaligning with the aim pursued. Foregrounding the physics aligns more closely with *scientific* than with *technological* understanding (for a discussion of their difference, see [9]). Insofar as it addresses technological understanding, it addresses the technical, design-type, which emphasises how physical phenomena interact with the technology’s structure. Often, such technical understanding—focused on a technology’s inner workings—is not essential for ethical reflection on its risks, benefits, and broader implications. Moreover, an overemphasis on physics, even in simplified form, may obscure rather than clarify quantum technologies, potentially hindering engagement and meaningful ethical discussions.

Stressing the primacy of functional understanding in enabling ethical engagement does not mean that technical understanding is irrelevant to such discussions. On the contrary, technical insights may substantially support ethical analysis. Some degree of technical understanding can be necessary for recognising functional capabilities and potential applications, and insight into a technology’s inner workings may illuminate specific issues, such as those related to direct safety, materials, or dependencies within a technology’s stack, that are less visible through other forms of understanding. Functional understanding, however, is indispensable for discussing a wide range of ethical issues and therefore constitutes the core epistemic requirement for ethical engagement. Technical understanding can complement this, but on its own is insufficient for a meaningful ethical discussion.

To foster meaningful ethical discussions about quantum technologies, especially in relation to their potential impacts, a functions-oriented approach to understanding is key. Rather than focusing on quantum mechanics, efforts to promote ethical engagement should focus on grasping how these technologies might function in practice. Recent literature appears to be moving in this direction, increasingly favouring functions-oriented discussions of quantum technologies over physics-focused explanations [4, 7, 20, 28, 29]. This is a much-needed development, now grounded in the theory of technological understanding, and deserves wider uptake both within and beyond academia. Importantly, fostering genuine functional understanding requires more than outlining possible applications: it involves enabling people to recognise the functional capabilities that underpin those applications and to grasp the scope of other potential uses.

Presenting functional understanding of quantum technologies as essential for ethical discussions has three implications. First, it raises *and* lowers the bar for ethical engagement at the same time: it establishes a necessary epistemic condition for engaging in such discussions, while simultaneously making this understanding more accessible by decoupling it from technical, physics-oriented expertise. Second, adopting a functions-oriented approach to fostering understanding of quantum technologies sets agendas: efforts to promote ethical engagement should focus on how these technologies might function in practice rather than on their inner workings. Where the functional capabilities of quantum technologies remain unclear, this points to the need to improve our understanding of what these technologies can and cannot do; and as long as this remains uncertain, ethical engagement should proceed cautiously when addressing questions of impact to avoid speculative overreach [30]. Third, prioritising functional over technical understanding suggests that those with expertise in quantum mechanics, despite their deep knowledge, are not automatically well positioned to engage in ethical reflection. They too must acquire a distinct kind of understanding focused on the potential functions and applications of the technology.

## Conclusion

The innovative potential of quantum technologies requires well-informed ethical discussions to ensure that their development and deployment is not only a technical success but also a net benefit for society and the planet. I have argued that effective ethical engagement is epistemically conditioned: it requires a certain degree of understanding of the technology. I used De Jong and De Haro’s account of technological understanding to clarify what such understanding entails: the ability to recognise how a technology can be used to achieve a practical aim. This perspective provides grounding and guidance for promoting understanding of quantum technologies to support ethical engagement.

Presenting technological understanding as a precondition for meaningful ethical discourse does not imply that ethicists or those involved in ethical discussions need to be technical experts, nor that technical experts are automatically well-positioned to engage in ethical reflection. Distinguishing between different types of technological understanding, I argued that one type in particular—*functional* understanding—is essential for ethical engagement. What matters is grasping the potential functionalities of quantum technologies to critically reflect on their ethical implications. Without such understanding, ethical discussions risk becoming overly speculative and detached from the realities of the technology.

Therefore, fostering meaningful ethical discussions about quantum technologies requires advancing an understanding of their potential functions. While this perspective raises an epistemic threshold for meaningful ethical discussion, it also makes it more accessible to a wider audience, fostering not only a well-informed but also a more inclusive ethical debate.

## Footnotes

1 This ability can be exercised both conceptually and practically.
